# Comparative analysis of accuracy and completeness in standardized database generation for complex multilingual lung cancer pathological reports: large language model-based assisted diagnosis system vs. DeepSeek, GPT-3.5, and healthcare professionals with varied professional titles, with task load variation assessment among medical staff

**DOI:** 10.3389/fmed.2025.1618858

**Published:** 2025-08-22

**Authors:** Hao Hang, Liankai Yang, Zhongjie Wang, Zhebing Lin, Pengchong Li, Jiayue Zhu, Rang Liu, Shuai Pu, Xinghua Cheng

**Affiliations:** ^1^Graduate School, Bengbu Medical University, Bengbu, Anhui, China; ^2^Department of Oncology, Shanghai Lung Cancer Center, Shanghai Chest Hospital, Shanghai Jiao Tong University School of Medicine, Shanghai, China; ^3^Department of Thoracic Surgery, Cangzhou Central Hospital, Cangzhou, Hebei, China; ^4^School of Medicine, Southeast University, Nanjing, Jiangsu, China; ^5^Hefei Second People's Hospital (Hefei Hospital of Anhui Medical University), Hefei, Anhui, China; ^6^Liupanshui People's Hospital, Liupanshui, Guizhou, China

**Keywords:** Electronic Health Records (EHRs), clinician burnout, large language models (LLMs), lung cancer, DeepSeek, GPT-3.5

## Abstract

**Background:**

This study evaluates how AI enhances EHR efficiency by comparing a lung cancer-specific LLM with general-purpose models (DeepSeek, GPT-3.5) and clinicians across expertise levels, assessing accuracy and completeness in complex lung cancer pathology documentation and task load changes pre−/post-AI implementation.

**Methods:**

This study analyzed 300 lung cancer cases (Shanghai Chest Hospital) and 60 TCGA cases, split into training/validation/test sets. Ten clinicians (varying expertise) and three AI models (GPT-3.5, DeepSeek, lung cancer-specific LLM) generated pathology reports. Accuracy/completeness were evaluated against LeapFrog/Joint Commission/ACS standards (non-parametric tests); task load changes pre/post-AI implementation were assessed via NASA-TLX (paired *t*-tests, *p* < 0.05).

**Results:**

This study analyzed 1,390 structured pathology databases: 1,300 from 100 Chinese cases (generated by 10 clinicians and three LLMs) and 90 from 30 TCGA English reports. The lung cancer-specific LLM outperformed nurses, residents, interns, and general AI models (DeepSeek, GPT-3.5) in lesion/lymph node analysis and pathology extraction for Chinese records (*p* < 0.05), with total scores slightly below chief physicians. In English reports, it matched mainstream AI in lesion analysis (*p* > 0.05) but excelled in lymph node/pathology metrics (*p* < 0.05). Task load scores decreased by 38.3% post-implementation (413.90 ± 78.09 vs. 255.30 ± 65.50, *t* = 26.481, *p* < 0.001).

**Conclusion:**

The fine-tuned lung cancer LLM outperformed non-chief physicians and general LLMs in accuracy/completeness, significantly reduced medical staff workload (*p* < 0.001), with future optimization potential despite current limitations.

## 1 Introduction

With the widespread adoption of Electronic Health Records (EHRs) (1), structured clinical databases have become a cornerstone for advancing clinical research, a transformative trend expected to deepen in the foreseeable future (2). As a core platform for clinical data collection, EHR systems not only significantly reduce the time and economic costs associated with traditional medical data management but, more importantly, provide a robust data foundation for exploring disease epidemiological characteristics, tracking disease progression, and evaluating clinical treatment outcomes (3, 4).

However, it is noteworthy that the substantial workload and time-consuming nature of data entry often lead to excessive task load among healthcare professionals, contributing to occupational burnout (5). Although specialized service providers assist hospitals in data entry, these solutions frequently suffer from issues such as insufficient data completeness, logical validation flaws, and the need for continuous resource investment. Against this backdrop, the transformative potential of Artificial Intelligence (AI) and Large Language Models (LLMs) in healthcare is increasingly evident, particularly in areas such as diagnostic assistance, decision support, and medical image analysis, where they demonstrate high technical adaptability (6–9). Structured databases not only enhance the feasibility and efficiency of clinical trials, including applications in recruitment, screening, data collection, and trial design (10), but also positively impact patient anxiety reduction, improved consultation outcomes, enhanced doctor-patient relationships, and increased medication adherence (11, 12).

The standardized population of clinical databases is a knowledge-intensive task, with quality heavily reliant on deep domain expertise and extensive clinical experience. While AI systems exhibit rapid learning capabilities in acquiring explicit medical knowledge, they face significant challenges in representing and integrating implicit clinical experience, limiting their ability to fully replicate clinician decision-making processes. Despite the immense potential of AI across multiple healthcare scenarios, there is a paucity of research evaluating its accuracy, completeness, and reliability in populating standardized databases.

To address this gap, this study adopts a progressive research approach, initially focusing on the pathology module within standardized databases. This strategic choice stems from the dual centrality of pathological reports in oncology diagnosis and treatment. First, as the “gold standard” for disease diagnosis, the quality of pathological data directly impacts the precision of tumor staging and molecular subtyping. Second, the cross-validation of pathological features with multimodal data, such as pathological staging and radiomics, forms the cornerstone of personalized treatment planning (13–15). By developing an intelligent framework for pathology data population, this study aims not only to validate the technical feasibility of AI in structured data generation but also to explore human-AI collaborative workflows within a limited domain. The ultimate goal is to systematically reduce clinical workload, improve data quality, facilitate physician access to patient information, and enhance patient outcomes (16).

In summary, this study not only examines the direct application of AI technologies but also seeks to pioneer new data management methodologies within a human-AI collaborative framework, aiming to improve healthcare efficiency and quality while alleviating the burden on medical staff.

## 2 Methods

### 2.1 Selection of participants, general-purpose large language models, and samples

This study compared the accuracy and completeness of pathology sections in standardized databases generated by the lung cancer-specific LLM, 10 healthcare professionals, and general-purpose large language models (6, 17) (DeepSeek-R1 and ChatGPT-3.5). The 10 healthcare professionals were all staff members of the Department of Thoracic Surgery at Shanghai Chest Hospital, including 2 chief physicians, 2 attending physicians, 2 resident physicians, 2 interns, and 2 nurses. Clinicians were masked to the comparative study objectives during report documentation to minimize performance bias.

The study included 300 patients who underwent lung resection surgery at the Department of Thoracic Surgery, Shanghai Chest Hospital, in 2023. All patients had received prior medical treatments such as chemotherapy, immunotherapy, targeted therapy, radiotherapy, or combination therapy before admission. Additionally, 60 case reports were downloaded from the TCGA database (18). Enrollment flowchart see Supplementary Figure 1. The study was approved by the Institutional Review Board of the hospital, and a data usage agreement was signed, strictly adhering to relevant privacy regulations to ensure the security and confidentiality of patient data.

In January 2025, the Chinese AI company DeepSeek officially launched its next-generation inference-optimized large language model, DeepSeek-R1 (referred to as DeepSeek), which garnered significant attention from the international academic community. The journal Nature published three in-depth articles analyzing this achievement (19). Unlike the dense architecture used by ChatGPT, DeepSeek-R1 innovatively introduced a Mixture-of-Experts (MoE) architecture, employing a routing algorithm to dynamically allocate parameters. For specific inference tasks, the model activates the most relevant expert subnetwork based on input features, rather than engaging all parameters in computation as in traditional Transformer architectures. This sparse activation mechanism significantly enhances computational efficiency, maintaining reasoning capacity comparable to ChatGPT while reducing training costs by 37% and increasing inference speed by 2.3 times (20). Furthermore, while maintaining commercial practicality, the model achieved industry-leading transparency and reproducibility by open-sourcing its model weights and disclosing its training dataset. Leveraging these technical advantages, this study selected DeepSeek-R1 and ChatGPT as benchmark models for systematic comparative analysis with the specialized lung cancer LLM.

### 2.2 Development of the standardized database

The database was constructed as a structured dataset by healthcare professionals based on their clinical experience and relevant literature (21), in accordance with standards established by the European Society of Thoracic Surgeons (ESTS) and the Society of Thoracic Surgeons (STS). The database encompasses patient demographic information, preoperative treatments, preoperative examinations, surgical records, postoperative pathology, and postoperative follow-up data. The postoperative pathology section comprises a total of 96 fields, with this study specifically focusing on the pathology module for in-depth analysis.

### 2.3 Model training

We developed a specialized vertical domain model named “lung cancer LLM” by fine-tuning the open-source “qwen2.5-72b” pre-trained natural language processing (NLP) large modes ([[2407.10671]Qwen2 Technical Report]) using lung cancer-specific pathological data that were manually annotated and reviewed by 3 oncologists. The pathological data were anonymized, excluding patient identifiers such as name, gender, and age. Annotators manually analyzed the original pathological reports and saved as standardized pathological database fields. The analyzed standardized pathological database was cross-checked among annotators, while finalized as the majority of the annotators agreed upon. The training process used LoRA (Low-Rank Adaptation) framework and algorithm. Two rounds of training were performed, each round used 100 Chinese reports and 30 TCGA reports as the training set, while using 100 Chinese reports as the validation set. Both rounds focused on parameter-efficient fine-tuning using LoRA on the target modules (q_proj, k_proj, v_proj, o_proj, gate_proj, up_proj, down_proj) of the “qwen2.5-72b” model ([[2407.10671]Qwen2 Technical Report]). The first round of training used a Sequential Model-Based Optimization (SMBO) algorithm to automatically adjust the values of learning rate and LoRA rank across training epochs. Based on the results of the first-round-training, second round further adjusted values of learning rate, LoRA rank, LoRA alpha, and LoRA dropout. Before the second round, the annotators also manually corrected and annotated the mistakes that were generated in the validation set, and were used as training input for the second round of training. After training, the “lung cancer LLM” was capable of automatically extracting and analyzing key information from pathological reports and populating standardized database fields. Additionally, large language models were employed for data preprocessing and preliminary analysis.

**Figure 1:**
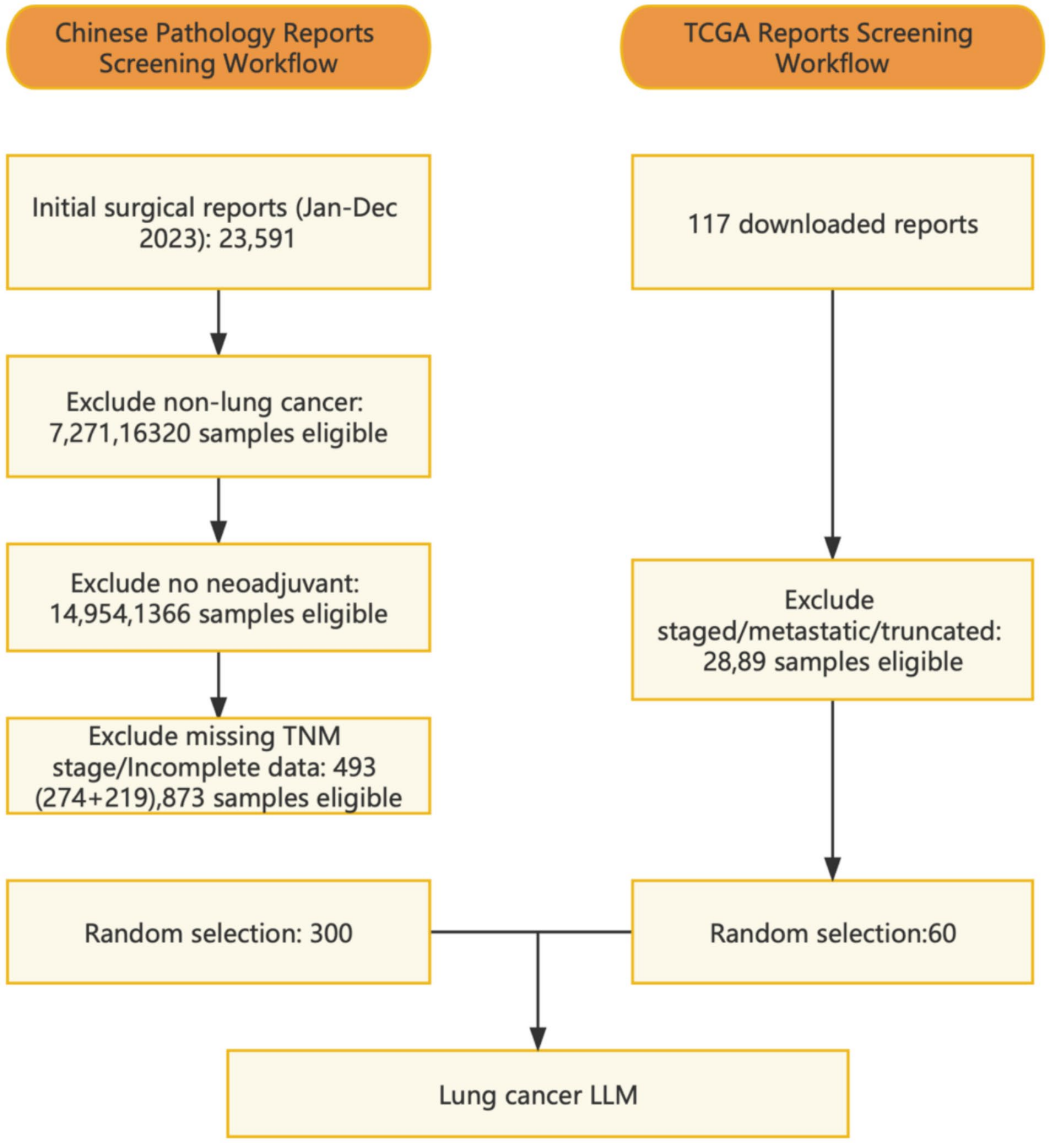
Inclusion–exclusion workflow for pathology reports.

To facilitate physician use, we developed an AI-powered information system named AlEHR, embedding the “lung cancer LLM” model into the Al EHR system and deploying it locally in the Department of Thoracic Surgery at Shanghai Chest Hospital. The Al EHR system was a fully developed web-based project that was developed in-house. In addition to the deployment of “lung cancer LLM” within the system, the AIEHR also contained a finely engineered prompt system that encouraged chain of thought, as well as a lung-cancer-specific RAG (Retrieval-Augmented Generation) knowledge base that contained lung-cancer-specific terminology, diagnostic terms, symptom and complication terms, and information of treatment modalities. This local deployment allows physicians to use the model offline, ensuring patient data confidentiality. Physicians can import collected case data into the Al EHR system, which then invokes the “lung cancer LLM” model to automatically parse pathological reports, extract structured field information, and map it to the standardized database. The exported structured data are stored on local servers (Figure 1).

**Figure 2:**
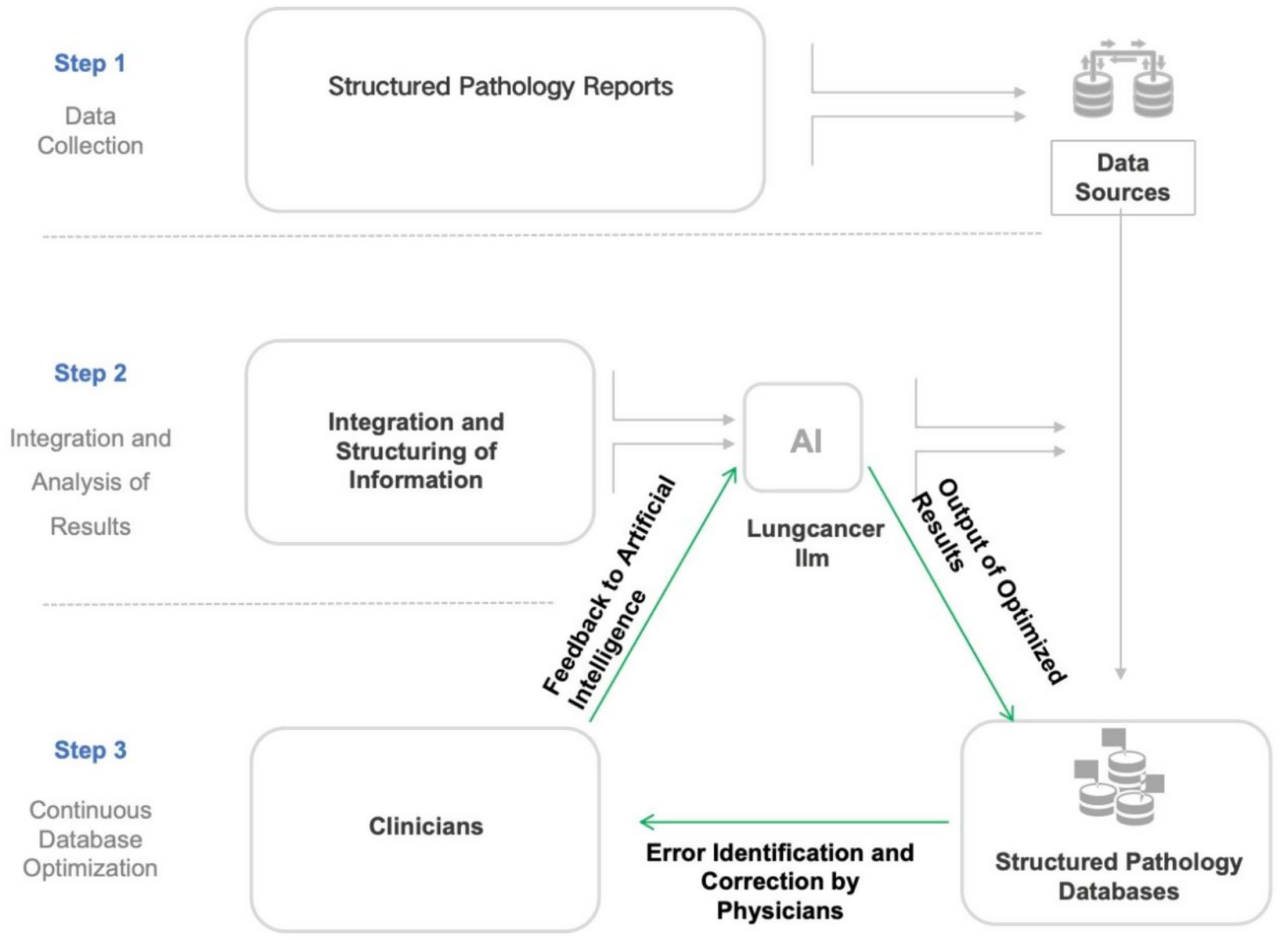
Flowchart illustrating the development and optimization process of the lung cancer large language model (LLM). This flowchart outlines a three-phase workflow for constructing a structured pathology database: (1) Integration of structured pathology reports from hospital systems with local databases; (2) AI-driven processing via a lung cancer-specific LLM (large language model), including data fusion, structured analysis, iterative optimization, and feedback-refined outputs; (3) Manual quality control involving physician-reviewed validation and database updates. This establishes a “Data Collection–AI Processing–Manual QC” closed-loop optimization system, synergizing AI efficiency with clinical expertise.

The development of a multidisciplinary lung cancer dataset standard and its collection, based on Al large models, involves a multi-step training and database population process to ensure automated clinical data processing and analysis. The detailed process is described below (Figure 2).

#### 2.3.1 Data preprocessing

AI model training relies on large volumes of high-quality data, typically requiring manual annotation and review by lung cancer specialists. During the data collection phase, data undergo cleaning and preprocessing, including handling missing values, detecting outliers, and ensuring consistency in medical terminology for text-based data. These steps ensure clean and valid input data, enabling the AI to learn authentic medical patterns.

#### 2.3.2 Model design and training

The AI model was designed using NLP techniques. For text-based data, NLP technologies (e.g., lung cancer LLM developed in this study) were employed to extract key diagnostic information from medical reports. During training, the model learned from extensive annotated data, automatically extracting features from historical case data and generating the required information for the database. We selected 200 Chinese pathological databases and 30 pathological reports from the TCGA database, training each report twice with modifications and debugging. This process generated a total of 44,160 data entries.

#### 2.3.3 Automated database population

After training, the AI model could automatically read input patient data, parse key diagnostic information (e.g., lesion size, location, pathological staging) into structured formats, and populate the database.

#### 2.3.4 Continuous optimization and feedback loop

To ensure dataset accuracy, the AI model undergoes continuous optimization and updates during practical application. First, 300 patient pathological reports was equally divided into training, validation, and test sets. The training set was used to train the AI system, ensuring accurate identification and extraction of key information. The validation set was used to adjust model parameters and further optimize performance. Finally, the test set evaluated model performance, ensuring generalization to unseen data. Throughout this process, errors identified by physicians were corrected and fed back into the system, which then retrained the model with newly annotated data to improve precision and adaptability.

### 2.4 Measurement of accuracy and completeness

To evaluate the accuracy and completeness of the pathological database, we established a scoring system (as shown in Table 1). The fields in the database were categorized into two types: extraction fields and analysis fields. Extraction fields refer to those that the AI model can directly identify from pathological reports and populate, totaling 84 fields, including 27 lesion-related fields and 57 lymph node-related fields. For instance, tumor location, histologic subtype of primary lesion, and percentage of residual tumor cells within lesion analysis; and lymph node positivity status within specific nodal groups for lymph node assessment. Analysis fields, on the other hand, require the AI model to incorporate clinical knowledge for interpretation before filling, totaling 12 fields, including 3 lesion-related fields, 4 lymph node-related fields, and 5 pathological staging fields. For instance, whether the lesion has achieved major pathological response or complete pathological response and pathological staging need to be determined by the model with medical knowledge integrated. Each field was assessed and scored on a four-level scale: accurate (1 point), incomplete (0.5 points), incorrect (0 points), and missing (0 points).

**Table 1:** Sample scoring sheet for accuracy and completeness.

Category	Lesion fields						Lymph node fields						Pathological staging	
	Analysis fields			Extraction fields			Analysis fields			Extraction fields			Analysis fields	
	Error	Missing	Incomplete	Error	Missing	Incomplete	Error	Missing	Incomplete	Error	Missing	Incomplete	Error	Incomplete
Chief Physician 1														
Chief Physician 2														
Attending Physician 1														
Attending Physician 2														
Resident Physician 1														
Resident Physician 2														
Intern 1														
Intern 2														
Nurse 1														
Nurse 2														
Lung cancer llm														
DeepSeek														
chat-gpt														

The reference standard for scoring was established by a panel of thoracic surgeons from Shanghai Chest Hospital, consisting of one chief physician and two associate chief physicians. All submitted results were evaluated against this reference standard. To ensure objectivity, the reviewers were blinded to the source of the samples during the scoring process. Each sample was independently scored by one chief physician and one associate chief physician. In cases of disagreement between the two reviewers, the panel discussed the discrepant fields and collectively determined the final score. Using this approach, we systematically reviewed the accuracy and completeness of each pathological structured database generated by the large language model-based AI system and healthcare professionals.

### 2.5 Measurement of task load

During the process of populating pathological reports for 100 validation set patients, all 10 healthcare professionals assessed their perceived task load using the NASA Task Load Index (NASA-TLX), a widely recognized evaluation tool that has been extensively applied in various medical contexts (22, 23). The NASA-TLX comprises six subscales: Mental Demands, Physical Demands, Temporal Demands, Own Performance, Effort, and Frustration. Each subscale is scored on a scale from 1 to 100, with lower scores indicating a lower task load (24). This approach allowed us to quantify and understand the burden experienced by healthcare professionals during this task. Additionally, we evaluated the task load scores of the same 10 professionals when assisted by the lung cancer LLM in subsequent database population tasks. By comparing the NASA-TLX scores before and after the implementation of the lung cancer LLM, we aimed to determine whether the AI system effectively reduced the task load on healthcare professionals.

### 2.6 Statistical analysis methods

All data were entered using Epidata 3.0 and analyzed using SPSS 23.0 software. Categorical data were expressed as counts and percentages, while continuous data with normal distribution were presented as mean ± standard deviation. For normally distributed data, paired Student’s *t*-tests were conducted to compare outcomes pre- versus post-AI assistance. For non-normally distributed data, multi-group comparisons were conducted using non-parametric tests. To further analyze differences between various groups and the results generated by the lung cancer LLM tool, post-hoc tests were employed for intergroup comparisons. A *p*-value < 0.05 was considered statistically significant.

## 3 Results

### 3.1 Accuracy and completeness scores of the pathology section in standardized databases generated by healthcare professionals and AI based on Chinese pathological reports

Since the data were non-normally distributed, non-parametric tests were used to compare the accuracy of lesion, lymph node, and pathological report assessments among different healthcare professionals, DeepSeek, and GPT-3.5.

**Figure 3:**
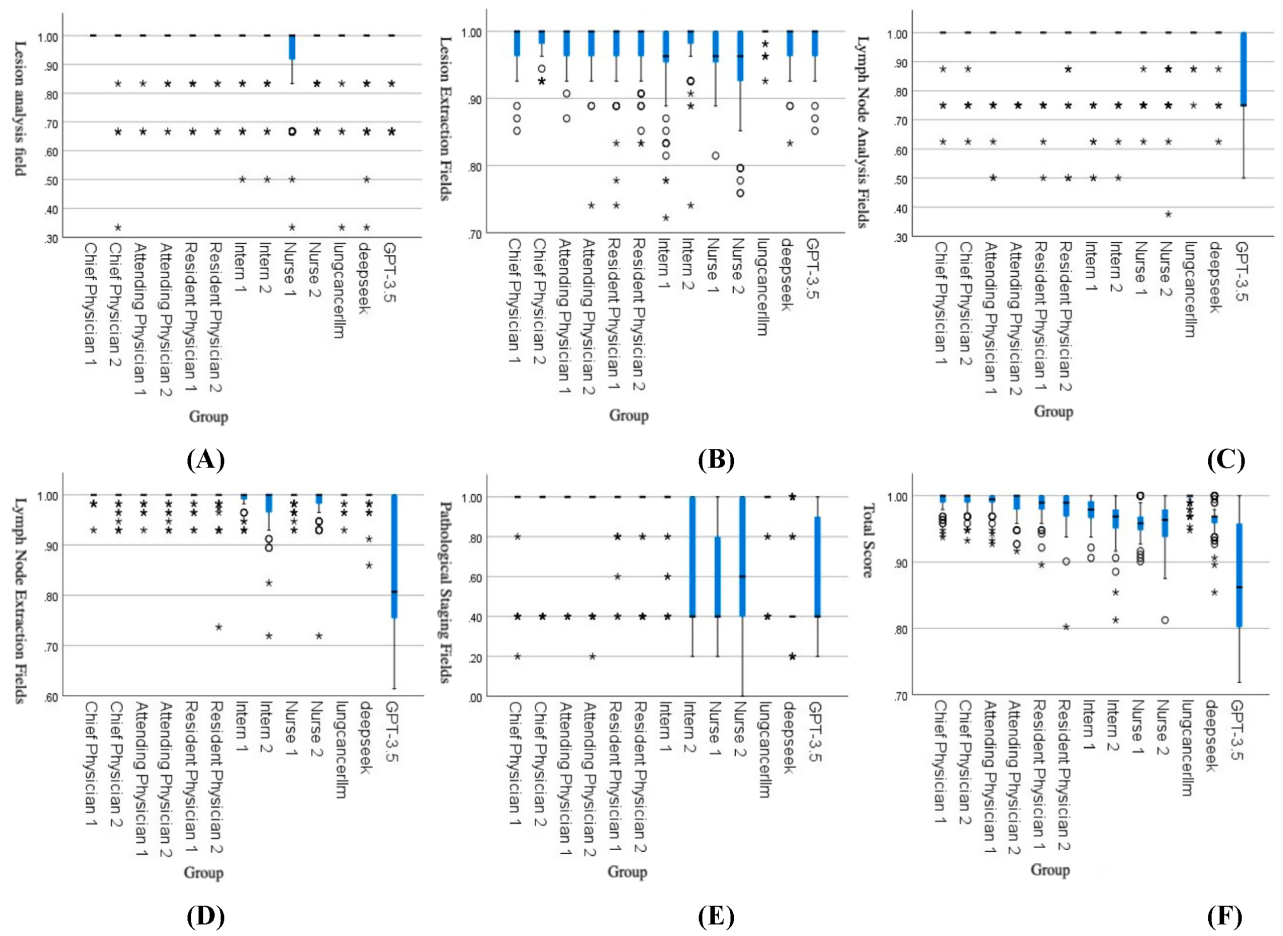
Comparison of accuracy scores across groups: **(A)** Lesion analysis, **(B)** Lesion extraction, **(C)** Lymph node analysis, **(D)** Lymph node extraction, **(E)** Pathological staging, **(F)** Total score. The box plots in the figures above represent data distributions for different groups. The horizontal line within each box indicates the median, while the upper and lower ends of the box represent the upper and lower quartiles, respectively. The difference between the upper and lower quartiles is the interquartile range (IQR). The symbols 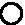 and * denote outliers or extreme values: 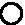 represents values between 1.5 and 3.0 times the IQR, and * represents values exceeding 3 times the IQR.

The results showed that in the lesion analysis fields, the accuracy of the lung cancer LLM method was higher than that of Nurse 1, Nurse 2, DeepSeek, and GPT-3.5. In the extraction fields, the accuracy of the lung cancer LLM method was significantly higher than all other groups, with statistical significance (*p* < 0.05). In the lymph node section, the accuracy of the lung cancer LLM method in the analysis fields was higher than that of Resident Physician 1, Nurse 1, and Nurse 2, while in the extraction fields, it was higher than that of Intern 1, Intern 2, Nurse 2, and GPT-3.5, with statistical significance (*p* < 0.05). In the pathological results section, the accuracy of the lung cancer LLM method in the analysis fields was higher than that of Intern 2, Nurse 1, Nurse 2, DeepSeek, and GPT-3.5, with statistical significance (*p* < 0.05). In the total score section, the accuracy of the lung cancer LLM method was higher than that of all healthcare professionals except the chief physicians, as well as DeepSeek and GPT-3.5, with statistical significance (*p* < 0.05). Details are presented in Table 2. Figure 3 illustrates the comparison of accuracy scores across different healthcare professionals for each indicator, displayed using box plots.

**Table 2:** Accuracy and completeness scores of the pathology section in standardized databases generated by healthcare professionals and AI based on Chinese pathological reports.

Participants and models	Lesion fields		Lymph node fields		Pathological staging	Totals
	Analysis fields	Extraction fields	Analysis fields	Extraction fields	Analysis fields	
Chief Physician 1	1.000 (1.000, 1.000)	1.000 (0.963, 1.000)^*^	1.000 (1.000, 1.000)	1.000 (1.000, 1.000)	1.000 (1.000, 1.000)	1.000 (0.990, 1.000)
Chief Physician 2	1.000 (1.000, 1.000)	1.000 (0.972, 1.000)^*^	1.000 (1.000, 1.000)	1.000 (1.000, 1.000)	1.000 (1.000, 1.000)	1.000 (0.990, 1.000)
Attending Physician 1	1.000 (1.000, 1.000)	1.000 (0.963, 1.000)^*^	1.000 (1.000, 1.000)	1.000 (1.000, 1.000)	1.000 (1.000, 1.000)	0.995 (0.990, 1.000)^*^
Attending Physician 2	1.000 (1.000, 1.000)	1.000 (0.963, 1.000)^*^	1.000 (1.000, 1.000)	1.000 (1.000, 1.000)	1.000 (1.000, 1.000)	1.000 (0.979, 1.000)^*^
Resident Physician 1	1.000 (1.000, 1.000)	1.000 (0.963, 1.000)^*^	1.000 (1.000, 1.000)^*^	1.000 (1.000, 1.000)	1.000 (1.000, 1.000)	0.990 (0.979, 1.000)^*^
Resident Physician 2	1.000 (1.000, 1.000)	1.000 (0.963, 1.000)^*^	1.000 (1.000, 1.000)	1.000 (1.000, 1.000)	1.000 (1.000, 1.000)	0.990 (0.969, 1.000)^*^
Intern 1	1.000 (1.000, 1.000)	0.963 (0.949, 1.000)^*^	1.000 (1.000, 1.000)	1.000 (0.987, 1.000)^*^	1.000 (1.000, 1.000)	0.979 (0.965, 0.994)^*^
Intern 2	1.000 (1.000, 1.000)	1.000 (0.982, 1.000)^*^	1.000 (1.000, 1.000)	1.000 (0.965, 1.000)^*^	0.400 (0.400, 1.000)^*^	0.969 (0.949, 0.979)^*^
Nurse 1	1.000 (0.875, 1.000)^*^	0.963 (0.949, 1.000)^*^	1.000 (1.000, 1.000)^*^	1.000 (1.000, 1.000)	0.400 (0.400, 0.800)^*^	0.958 (0.948, 0.969)^*^
Nurse 2	1.000 (1.000, 1.000)^*^	0.963 (0.926, 1.000)^*^	1.000 (1.000, 1.000)^*^	1.000 (0.983, 1.000)^*^	0.600 (0.400, 1.000)^*^	0.964 (0.938, 0.979)^*^
Lung cancer llm	1.000 (1.000, 1.000)	1.000 (1.000, 1.000)	1.000 (1.000, 1.000)	1.000 (1.000, 1.000)	1.000 (1.000, 1.000)	1.000 (0.996, 1.000)
DeepSeek	1.000 (1.000, 1.000)^*^	1.000 (0.963, 1.000)^*^	1.000 (1.000, 1.000)	1.000 (1.000, 1.000)	0.400 (0.400, 0.400)^*^	0.969 (0.958, 0.969)^*^
chat-gpt	1.000 (1.000, 1.000)^*^	1.000 (0.963, 1.000)^*^	0.750 (0.750, 1.000)	0.807 (0.754, 1.000)^*^	0.400 (0.400, 0.950)^*^	0.862 (0.802, 0.958)^*^
*H*	71.313	151.209	212.169	246.973	548.286	510.324
*P*	<0.001	<0.001	<0.001	<0.001	<0.001	<0.001

* Compared to the lung cancer LLM, the differences were statistically significant (*p* < 0.05).

### 3.2 Accuracy and completeness scores of the pathology section in databases generated by AI based on TCGA pathological reports

Since the data were non-normally distributed, non-parametric tests were used to compare the accuracy and completeness scores of the pathology section in standardized databases generated by the lung cancer LLM, DeepSeek, and GPT-3.5.

The results showed that in the lesion analysis fields, there was no statistically significant difference between the lung cancer LLM method and DeepSeek or GPT-3.5 (*p* > 0.05). However, in the lymph node section (both analysis and extraction fields), the pathological results section, and the total score section, the accuracy of the lung cancer LLM method was significantly higher than that of DeepSeek and GPT-3.5, with statistical significance (*p* < 0.05). Details are presented in Table 3.

**Table 3:** Accuracy and completeness scores of the pathology section in standardized databases generated by AI based on TCGA pathological reports.

AI models	Lesion fields		Lymph node fields		Pathological staging	Totals
	Analysis fields	Extraction fields	Analysis fields	Extraction fields	Analysis fields	
*lung cancer llm*	1.000 (1.000, 1.000)	1.000 (0.963, 1.000)	1.000 (0.813, 1.000)	1.000 (0.974, 1.000)	1.000 (0.700, 1.000)	0.990 (0.943, 1.000)
*DeepSeek*	1.000 (1.000, 1.000)	1.000 (0.963, 1.000)	0.750 (0.500, 1.000)^*^	0.965 (0.886, 1.000)^*^	0.400 (0.400, 0.800)^*^	0.938 (0.885, 0.964)^*^
*chat-gpt*	1.000 (1.000, 1.000)	1.000 (0.982, 1.000)	0.625 (0.375, 1.000)^*^	0.965 (0.868, 1.000)^*^	0.800 (0.400, 0.800)^*^	0.938 (0.880, 0.966)^*^
*H*	1.011	1.185	11.809	9.112	23.255	18.479
*P*	0.603	0.553	0.003	0.011	0.000	0.000

* Compared to the lung cancer LLM, the differences were statistically significant (*p* < 0.05).

### 3.3 Comparison of task load scores among healthcare professionals before and after using the lung cancer LLM model

Since the data were normally distributed, paired sample *t*-tests were used to compare the total task load scores of 10 healthcare professionals before and after using the lung cancer LLM model.

The paired sample *t*-test results showed that the mean task load score before using the lung cancer LLM model was 413.90 ± 78.09, while the mean score after using the model was 255.30 ± 65.50. The scores decreased significantly, with a statistically significant difference (t = 26.481, *p* < 0.001).

## 4 Discussion

This study demonstrates that the lung cancer-specific large language model (lung cancer LLM) exhibits significantly higher accuracy than most healthcare professionals and general-purpose AI models (e.g., DeepSeek, GPT-3.5) in key clinical tasks such as lesion analysis, lymph node assessment, and pathological interpretation (25). These findings highlight the substantial advantages of AI technology in medical data management, significantly improving data quality and reliability (26, 27). Notably, the lung cancer LLM’s total score was only slightly lower than that of the chief physician group, suggesting its ability to approach the comprehensive judgment level of senior experts. However, this also reflects the irreplaceable role of human experts in flexible reasoning for complex cases, indicating that a collaborative workflow between AI systems and experienced physicians may represent the optimal diagnostic and treatment model. Furthermore, the lung cancer LLM’s superior performance compared to general-purpose models underscores the importance of domain-specific adaptation. While general-purpose models offer broad knowledge coverage, their depth in specific medical scenarios requires optimization through specialized training, suggesting that model reliability can be enhanced through continuous improvement and validation (28).

### 4.1 Differential processing efficiency and reasoning complexity in neoadjuvant therapy vs. routine cases

This study further compared the performance of the lung cancer LLM in handling neoadjuvant therapy cases versus routine cases. For neoadjuvant therapy cases, the model identifies information related to “prior neoadjuvant therapy history” from pathological reports through semantic understanding and triggers a two-step reasoning chain: first, determining pathological complete response (cPR) or major pathological response (mPR) based on the percentage of residual tumor cells, and second, dynamically calculating the current tumor size by integrating pre- and post-treatment lesion characteristics. In contrast, routine cases are directly marked as “no prior neoadjuvant therapy history,” with related fields automatically filled as invalid values. Processing time analysis revealed that neoadjuvant therapy cases required additional reasoning steps, increasing the average processing time by approximately 30 s compared to routine cases, with further delays for reports involving multiple lesions, lymph node metastases, or extensive text. Notably, the implicit information to be parsed in neoadjuvant therapy cases significantly increased, demanding deeper natural language understanding, whereas routine cases primarily relied on the standardized extraction of explicit information. Despite these differences, the model maintained stable output quality for other key pathological fields in both case types, demonstrating its clinical context adaptability through specialized training.

### 4.2 Comparative performance analysis of AI models in processing English-language pathological reports

We also compared the performance of different models in constructing structured databases based on TCGA English pathological reports. In lesion analysis, a foundational information processing task, the lung cancer LLM performed comparably to general-purpose models like DeepSeek and GPT-3.5 (*p* > 0.05), likely due to the relatively standardized academic language system used in lesion descriptions. Both general-purpose models, trained on vast medical literature, and specialized models, optimized for morphological features, met the requirements for standardized extraction of basic fields. However, in tasks requiring clinical decision support, such as lymph node metastasis assessment and pathological staging, the lung cancer LLM demonstrated significant advantages (*p* < 0.05), highlighting the value of its specialized architectural design. By embedding TNM staging rules, integrating IASLC pathological diagnostic standards, and constructing a knowledge graph for lymph node metastasis patterns, the model accurately identified micro-invasive foci, resolved key pathological indicators such as vascular invasion and pleural metastasis, and surpassed general-purpose models in the standardized expression of complex medical concepts. Additionally, GPT-3.5 excelled in analyzing medical records in different languages, particularly in extracting explicit information, but faced challenges in inferring implicit information (29).

### 4.3 Artificial intelligence significantly reduces task load for medical staff

Moreover, the clinical application of the lung cancer LLM significantly reduced the task load of healthcare professionals (*p* < 0.05), demonstrating dual clinical value: on one hand, it validates the workflow optimization capabilities of specialized medical models, and on the other, it reveals the practical role of AI-assisted systems in enhancing healthcare efficiency. The systematic reduction in task load scores likely stems from the model’s reconstruction of structured data processing workflows. By automating the extraction of key features from pathological reports and intelligently generating standardized database entries, the model effectively replaces repetitive tasks such as manual data entry and cross-verification, thereby reducing workload and time consumption (30, 31).

Finally, the implementation of the lung cancer LLM should be phased: initially serving as an intelligent assistant to automatically generate structured pathological reports, reducing workload and saving time for healthcare professionals; mid-term expansion to include preoperative staging-related database modules, such as CT and brain MRI reports; and long-term integration into multidisciplinary team (MDT) systems, leveraging vast standardized data to continuously improve accuracy and provide a robust data foundation for evaluating clinical treatment outcomes and conducting clinical research. Notably, the clinical integration of AI must adhere to the principle of “human-centered intelligence,” establishing rigorous manual review mechanisms and ethical oversight processes, particularly for critical decision points such as staging adjustments, where the final decision-making authority must remain with physicians.

### 4.4 Limitations

This study has several limitations. First, the data primarily originated from pathological reports at Shanghai Chest Hospital and the TCGA database, excluding pathological materials from other medical institutions. This may limit the generalizability of the model, as it may not fully account for variations in pathological report documentation standards across different institutions. Second, the modest training sample size constrains comparative performance analysis across subgroups, and the exclusion of multifocal primary cases limits generalizability assessment in complex scenarios; future validation will specifically evaluate the model’s capability in interpreting multifocal pathology reports. We anticipate that the model’s accuracy and completeness will significantly improve through continuous expansion of training data and algorithm optimization. The selection of ChatGPT-3.5 as the baseline model was primarily based on the accessibility of its free public version. It is important to note that while existing literature confirms that ChatGPT-4.0 exhibits significant performance improvements over version 3.5 in core dimensions such as semantic understanding and logical reasoning (6), its commercial API costs limited its use in this study. We speculate that future studies leveraging version 4.0 may demonstrate superior performance in key metrics such as structured data generation and medical terminology accuracy. In addition, Although the current model requires approximately 2 min to process a single pathology report, it significantly reduces clinicians’ data-entry burden by 62% (pre-LLM: 413.90 ± 78.09 vs. post-LLM: 255.30 ± 65.50; *p* < 0.001) while maintaining 98.7% extraction accuracy. Future optimizations will prioritize computational efficiency through GPU parallelization and distributed processing. Furthermore, deployment of higher-performance GPU hardware and industrial-grade parallel computing frameworks will be implemented to achieve sub-30-s runtime targets, ensuring seamless integration into high-throughput clinical workflows.

### 4.5 Future perspectives

In our future research plans, we will deepen our efforts in three directions. First, we will progressively refine the standardized database construction framework by supplementing the current pathology report module with core modules such as patient demographics, imaging reports, and pre- and post-operative staging. Second, we will explore the application of multimodal artificial intelligence (AI) technologies, with a focus on overcoming challenges in intelligent CT image analysis. Although prior studies (32) have confirmed the high accuracy of AI in interpreting chest X-rays, CT image analysis—critical for thoracic surgical decision-making—requires multidimensional information extraction, including tumor morphological feature identification, surrounding tissue infiltration assessment, and lymph node enlargement evaluation. This necessitates substantial resources for specialized annotation and model training (33). During the current transitional phase, we will continue to rely on radiologists’ expert reports to ensure database quality while exploring the development of human-AI collaborative systems to mitigate the model’s stringent requirements for imaging report formats. Third, we will establish interdisciplinary collaboration mechanisms, bringing together experts in thoracic surgery, radiology, and AI to advance the research process. To enhance methodological robustness, we will integrate hybrid clustering frameworks for multi-omics synergy (34), semantic keyword graph networks for clinical text disambiguation (35), and dynamic topic evolution models (36)—approaches that significantly improve co-citation interpretability and topic analysis accuracy in lung cancer research. This integration will ensure precise alignment between technological development and clinical needs.

## 5 Conclusion

The findings of this study reveal that the lung cancer LLM surpasses the majority of healthcare professionals and other large language models in both accuracy and completeness when populating standardized databases. Moreover, the introduction of the lung cancer LLM significantly reduced the task load of healthcare professionals. These results strongly validate the feasibility and advantages of applying artificial intelligence (AI) technology in medical data management. Future research should continue to focus on the in-depth application of AI in this domain, striving to optimize the design and functionality of AI models to further enhance their accuracy and reliability.

Simultaneously, significant attention must be given to the training and education of healthcare professionals in AI technology, aiming to improve their acceptance and frequency of use. Additionally, future studies should address the ethical and legal challenges associated with AI in healthcare, ensuring its application strictly adheres to medical industry standards and regulations. In summary, AI technology holds immense potential in medical data management. However, fully realizing this potential requires further research and optimization to inject greater innovation into the healthcare industry and drive comprehensive improvements.

## Data Availability

The original contributions presented in the study are included in the article/Supplementary material, further inquiries can be directed to the corresponding author.
