# Supplementary material for: Comparative analysis of accuracy and completeness in standardized database generation for complex multilingual lung cancer pathological reports: large language model-based assisted diagnosis system vs. DeepSeek, GPT-3.5, and healthcare professionals with varied professional titles, with task load variation assessment among medical staff
Source: Front Med (Lausanne). 2025 Aug 22;12:1618858. doi: 10.3389/fmed.2025.1618858 (PMC12411430; doi:10.3389/fmed.2025.1618858)
Supplement: Supplementary file 1 [file Supplementary_file_1.docx]

Supplementary Material

# Supplementary Figures
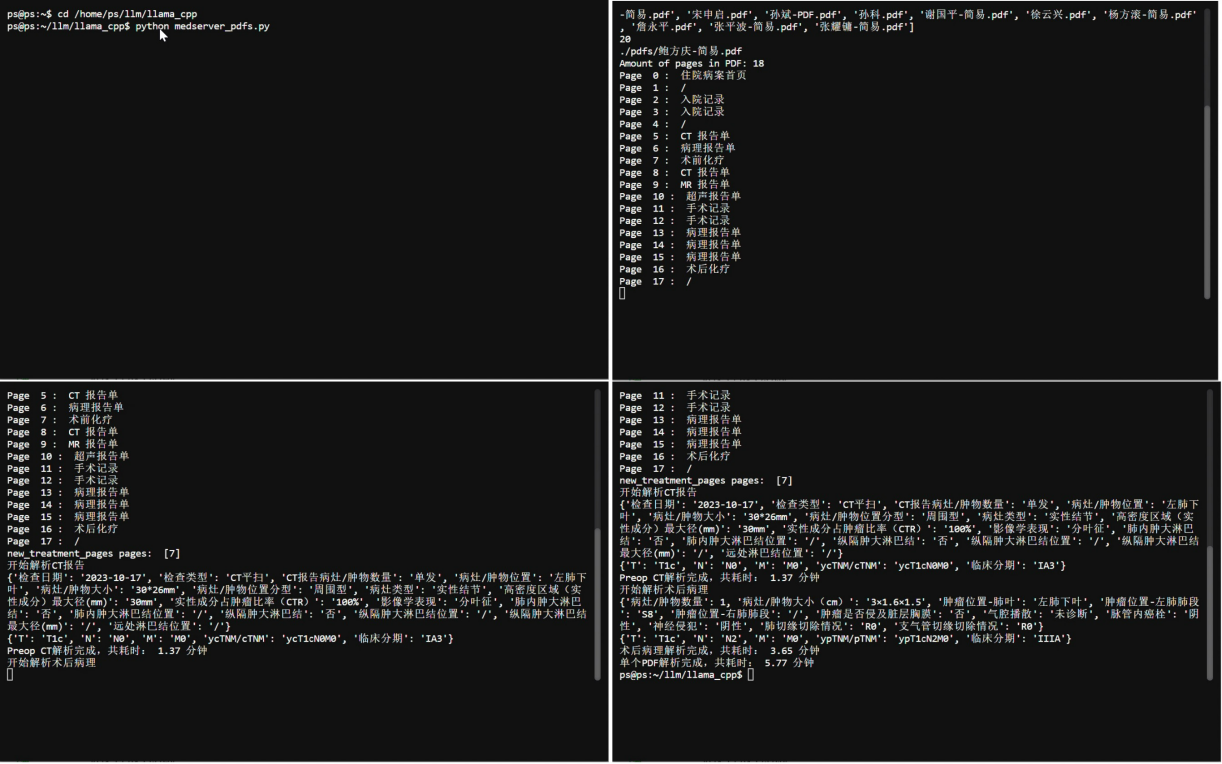


# **Supplementary Figure 1.** Utilizing multimodal large-scale models, OCR, and NLP technologies to achieve the extraction, recognition, and analysis of standardized medical data.

#
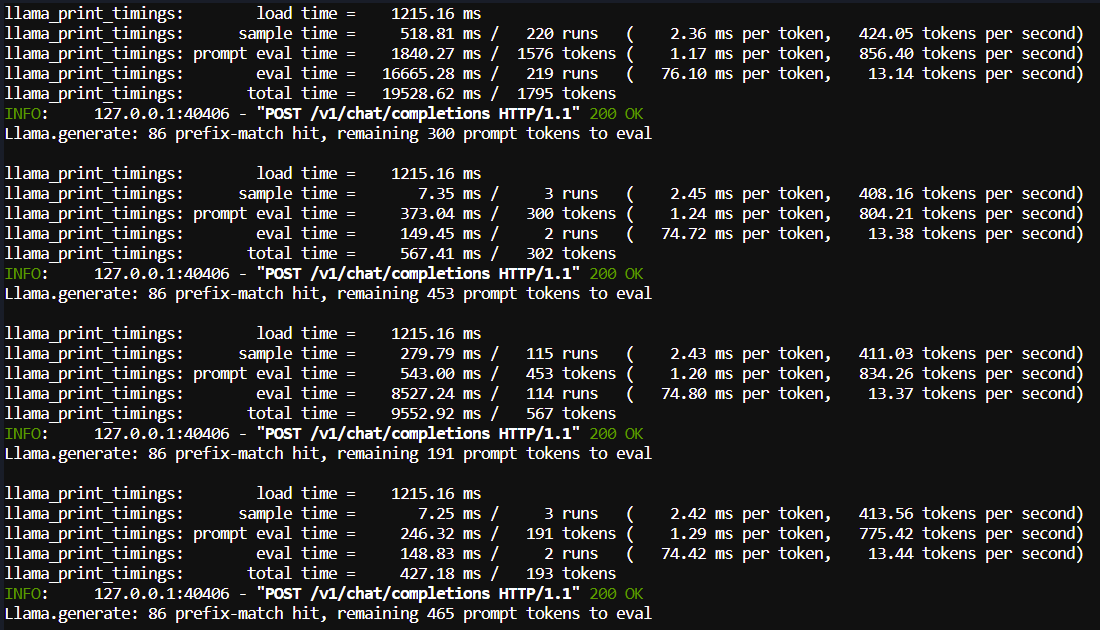


**Supplementary Figure 2.** In multimodal medical data analysis, key information is extracted and structured.
